# Intact cord resuscitation versus early cord clamping in the treatment of depressed newborn infants during the first 10 minutes of birth (Nepcord III) – a randomized clinical trial

**DOI:** 10.1186/s40748-019-0110-z

**Published:** 2019-08-29

**Authors:** Ola Andersson, Nisha Rana, Uwe Ewald, Mats Målqvist, Gunilla Stripple, Omkar Basnet, Kalpana Subedi, Ashish KC

**Affiliations:** 1 Department of Clinical Sciences Lund, Pediatrics/Neonatology, Skane University Hospital, Lund University, SUS, Barn- & Ungdomssjukh. Avd. Ped, 221 85 Lund, Sweden; 2 International Maternal and Child Health, Department of Women’s and Children’s Health, Uppsala University, Uppsala, Sweden; 3 Wass Medicin, Lund, Sweden; 4 Golden Community, Kathmandu, Nepal; 5 Paropakar Maternity and Women’s Hospital, Kathmandu, Nepal

**Keywords:** Resuscitation, Umbilical cord, Cord clamping, Term newborn, Pulse oximetry, Apgar score

## Abstract

**Background:**

Experiments have shown improved cardiovascular stability in lambs if umbilical cord clamping is postponed until positive pressure ventilation is started. Studies on intact cord resuscitation on human term infants are sparse. The purpose of this study was to evaluate differences in clinical outcomes in non-breathing infants between groups, one where resuscitation is initiated with an intact umbilical cord (intervention group) and one group where cord clamping occurred prior to resuscitation (control group).

**Methods:**

Randomized controlled trial, inclusion period April to August 2016 performed at a tertiary hospital in Kathmandu, Nepal. Late preterm and term infants born vaginally, non-breathing and in need of resuscitation according to the ‘Helping Babies Breathe’ algorithm were randomized to intact cord resuscitation or early cord clamping before resuscitation. Main outcome measures were saturation by pulse oximetry (SpO_2_), heart rate and Apgar at 1, 5 and 10 minutes after birth.

**Results:**

At 10 minutes after birth, SpO_2_ (SD) was significantly higher in the intact cord group compared to the early cord clamping group, 90.4 (8.1) vs 85.4 (2.7) %, *P* < .001). In the intact cord group, 57 (44%) had SpO_2_ < 90% after 10 minutes, compared to 93 (100%) in the early cord clamping group, *P* < 0.001. SpO_2_ was also significantly higher in the intervention (intact cord) group at one and five minutes after birth. Heart rate was lower in the intervention (intact cord) group at one and five minutes and slightly higher at ten minutes, all significant findings. Apgar score was significantly higher at one, five and ten minutes. At 5 minutes, 23 (17%) had Apgar score < 7 in the intervention (intact cord) group compared to 26 (27%) in the early cord clamping group, *P* < .07. Newborn infants in the intervention (intact cord) group started to breathe and establish regular breathing earlier than in the early cord clamping group.

**Conclusions:**

This study provides new and important information on the effects of resuscitation with an intact umbilical cord. The findings of improved SpO_2_ and higher Apgar score, and the absence of negative consequences encourages further studies with longer follow-up.

**Trial registration:**

Clinicaltrials.gov NCT02727517, 2016/4/4

**Electronic supplementary material:**

The online version of this article (10.1186/s40748-019-0110-z) contains supplementary material, which is available to authorized users.

## Background

Two thirds of a million term newborns die worldwide due to intrapartum-related events, the majority due to asphyxia and a disproportionate amount occurring in low-resource countries [1, 2]. An estimated additional 413,000 infants will develop neonatal encephalopathy after an intrapartum event, resulting in moderate or severe neurodevelopmental impairment. In the Global Burden of Disease 2010 study, intrapartum-related conditions comprised 50.2 million disability adjusted life years [3]. A high priority goal in the global community as recognized by the World Health Organization is prevention of stillborn and newborn mortality; this has resulted in initiatives such as Every Newborn Action Plan [4, 5].

Refined and revised resuscitation guidelines, recommendations, and algorithms during the past decade give health providers evidence-based tools and instructions that may help reduce morbidity and mortality. Adoption and implementation of the International Liaison Committee on Resuscitation (ILCOR) recommendations vary, and initiatives such as Neonatal Resuscitation Program and Helping Babies Breathe have identified the necessity of training and simulation needed to sustain change [6–8].

A non-interventional approach has gained interest and adoption, namely umbilical cord clamping (CC) that is delayed. In a study involving 456 infants, Chaparo et al. could show that by delaying CC until 2 minutes, iron stores were improved at 6 months of age [9]. Furthermore, we have shown that delaying CC until three minutes is associated with less anemia and improved development at 12 months of age in population in high-risk of iron deficiency and with improved fine motor function and social behavior at 4 years of age in a high income country [10–12]. However, this simple, and cost-free evidence-based practice is not widely adopted, although supported by several stakeholders [13].

In most studies, non-vigorous, non-breathing newborns have been excluded. ILCOR recommendations from 2015 states that there is insufficient evidence to recommend an appropriate time for clamping the cord in babies who require resuscitation at birth [6]. The American College of Obstetricians and Gynecologists’ Committee on Obstetric Practice declare the need of immediate resuscitation of the newborn to be an indication for immediate cord clamping [14]. In practice, this means that when children are born with reduced vitality, the umbilical cord will be clamped and cut off as soon as possible and the child transferred to a surface prepared for resuscitation, either in the ward or in an adjoining room.

Recent animal experiments have shown if CC occurs before gas exchange of the lung has been established, the physiological circulation can be severely compromised [15, 16]. These studies have led to proposals for newborns to be resuscitated without clamping the umbilical cord, i.e. resuscitation measures including clearing the airway, stimulating, and, if necessary, ventilation, are initiated while the child still has an intact umbilical cord [17, 18].

In humans, the transfused blood from the placenta during the first few minutes can increase overall blood volume with improved cardiac output and better perfusion in both central and peripheral tissues. If placenta circulation is allowed to continue there may be mitigation of the hypoxia, hypoglycemia and acidosis that occurs during a period of asphyxia [19]. An observational study has shown that delayed CC of vital newborn infants resulted in better oxygenation of the blood during the first 10 minutes [20]. A feasibility randomized trial has shown that newborns predicted to be at risk of resuscitation had greater cerebral oxygen at 12 h of age when CC was at five minutes compared to CC within one minute [21].

This area of research is new, and more randomized controlled trials of delayed CC after onset of ventilation are needed to investigate if this practice will result in short-term benefits as well as improved long-term neurodevelopmental outcomes [22]. As the main burden of neonatal mortality due to intra-partum events lies with low resource settings in South Asia and sub-Saharan Africa, we chose to implement our study in Nepal [3].

The purpose of our study was to evaluate differences in clinical outcomes in non-breathing newborns in need of resuscitation between groups, one where resuscitation is initiated prior to CC (intervention group) compared to a group where CC occurs prior to resuscitation (control group).

## Methods

### Study design

We conducted a randomized controlled trial (parallel group study with 1:1 randomization) of pregnancies, finalizing inclusion only after birth of the baby. Ethical approval was obtained from the Nepal Health Research Council, reg. no. 92/2015. Trial registration: Clinicaltrials.gov NCT02727517.

### Setting

The study was performed at the low risk-Maternal and Neonatal Service Centre (MNSC) at Paropakar Maternity and Women’s Hospital in Kathmandu, Nepal. Part of the research team had performed an intervention project introducing a simplified algorithm for neonatal resuscitation, Helping Babies Breathe (HBB) at the hospital [23]. The HBB algorithm is routine at the hospital and emphasizes that within the first minute after birth, “The Golden Minute”, a baby should breathe or receive positive-pressure ventilation. All babies who do not cry after drying should have their airways cleared and receive stimulation to breathe, followed by positive-pressure ventilation if necessary [4]. Before the study all staff underwent a recapitulation of the HBB algorithm including training sessions with the modified algorithm performing intact cord resuscitation. The research team members consisted of trained nurse-midwives with experience in clinical research, five on duty during all hours: one at the antenatal care unit, three at the delivery unit and one at the postnatal care unit. A 10-day training program was provided on selection criteria of women, taking consent, Apgar (including breathing) assessment and placement of the neonatal Masimo**©** pulse oximeter probe (Masimo Corporation, Switzerland).

### Inclusion and consent

The hospital criteria for admission to MNSC were: uncomplicated pregnancies, no complication at admission, healthy mothers (no clinical history of hypertension, infection, diabetes, or chronic medical condition), expected vaginal delivery, gestational age between 34 and 41 weeks, and singleton pregnancy.

Women were eligible to participate in the study if they were assigned to MNSC. Mothers admitted to MNSC were informed and asked for consent. Four research team members were present at admission and at the delivery department at all hours and days of the week. Newborns were eligible and included if they had reached a gestational age ≥ 33 weeks, were in need of resuscitation according to the HBB algorithm, that is: no breathing despite thorough drying and additional stimulation within 30 s after birth [24]. Newborns not in need of resuscitation were included in a parallel study, Nepcord II [25].

Exclusion criteria: Monochorionic twins (from an ultrasound scan) or clinical evidence of twin-twin transfusion syndrome, triplets or higher order multiple pregnancy, and fetuses with known congenital malformation.

### Randomization

Sequentially numbered, opaque envelopes containing the allocation were prepared by one of the authors (AKC) using a random digit generated list. AKC had no further clinical involvement in the trial.

**Fig. 1:**
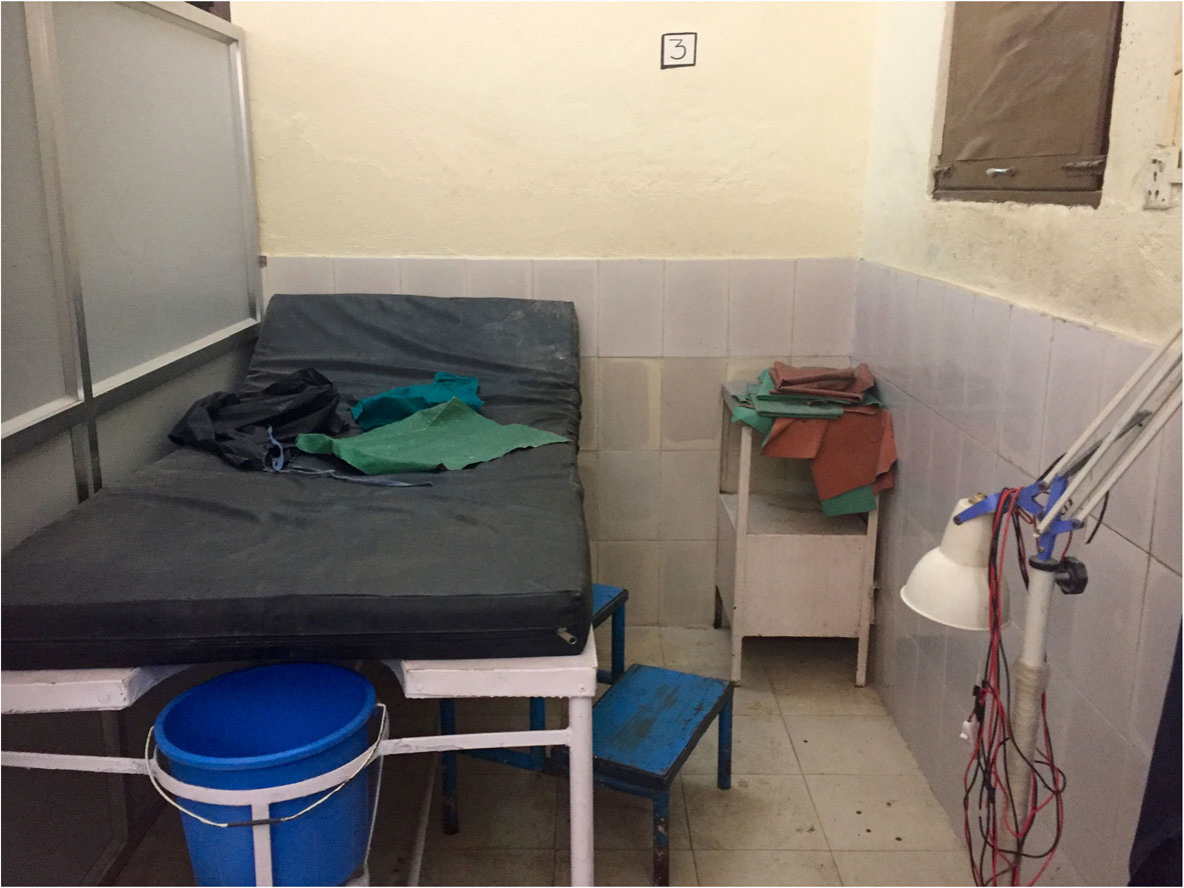
Image of a bed in the delivery department

**Fig. 2:**
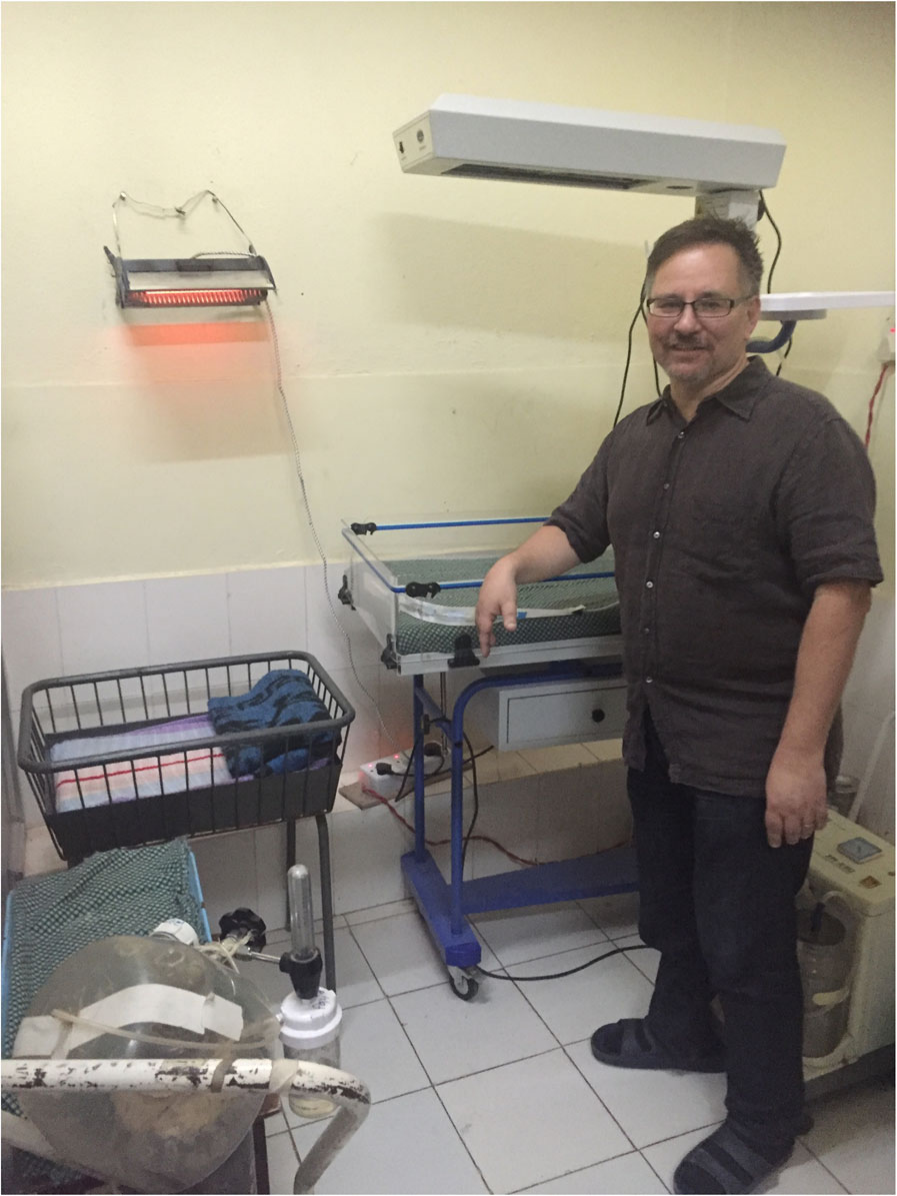
Image of the resuscitation table in a room next to the delivery room. The corresponding author standing beside it

Randomization was performed as follows: resuscitation with an intact umbilical cord close to the mother in her bed (Fig. 1), or standard care (resuscitation according to the HBB algorithm including early cord clamping) at a designated area, on a resuscitation table in a room next to the delivery room (Fig. 2). Delay in cord clamping was instructed to be at least 180 s in the intact cord group. Allocation was decided when birth was imminent, the research team member opened the next opaque numbered envelope and informed the staff managing the delivery on handling strategy of the allocated group.

### Blinding

Due to the nature of the intervention and the close proximity in time to outcome measures, blinding was not possible.

### Data collection

When the child was born, 2–3 research team members collaborated to start a timer, apply a pulse oximetry sensor on the right hand/arm of the newborn infant, and record time of first breath, time for regular breathing, time for cord clamping as well as Apgar score.

### Primary outcome

Saturation by pulse oximetry (SpO_2_) at 10 min after birth.

### Secondary outcome

- SpO_2_ at five minutes after birth
- Time to reach > 90% in SpO_2_
- Newborn heart rate at one, five and ten minutes after birth measured by pulse oximeter.
- Apgar score at one, five and ten minutes after birth
- Timing of recorded first breathing effort and established regular breathing
- Rectal temperature at 10–20 min
- Bilirubin at discharge from hospital
- Health status the day after birth (12–36 h after birth) – Alive or deceased

Background information regarding the women and pregnancies were collected from the hospital records by a research team member. All data were transferred to an individual datasheet. Data sheets were checked for quality and stored in a locked room at the hospital until the end of the study. Data were then entered into a database and used for statistical analyses.

### Sample size

A power analysis shows that a group size of 99 would allow us to find a difference of 2% in SpO_2_ between groups at 10 minutes after birth with a power of 80% and a significance level of 0.05, assuming a mean saturation (SD) of 96% (5) in the delayed cord clamping group [26]. Allowing for an attrition of 20%, we planned to include 120 participants in each group.

### Statistical analysis

For group comparisons of continuous variables, we used unpaired Student’s t-test for variables with normal distribution and Mann-Whitney U test for variables with skewed distribution. Categorical variables were compared between groups using Fisher’s exact test.

We calculated the numbers needed to treat, relative risk reduction, and their confidence intervals using the web based JavaStat calculator. A *p*-value < 0.05 was considered significant.

All analyses were made on an intention to treat basis as prespecified in the study protocol.

We performed all analyses for the primary and secondary outcomes, including only cases handled according to allocation (per protocol). Results are reported in Table 3.

### Sensitivity analysis

To examine if adjusted analysis would yield results different from unadjusted analysis, we analyzed the correlation coefficients between baseline data that was not randomly distributed between treatment groups with primary and secondary outcomes with Spearman’s rank correlation.

### Auxiliary analysis

Due to uneven group size after randomization and a large protocol violation rate in the intact cord group, we also chose to do alternate analysis including only infants where resuscitation was performed with bag and mask, reported in Table 4. An ANOVA analysis including post-hoc multiple comparisons comparing the infants divided into three groups, intact cord resuscitation per protocol (C ≥ 180 s), intact cord resuscitation protocol violation (CC < 180 s) and early CC was also performed [see Additional file 1].

## Results

**Fig. 3:**
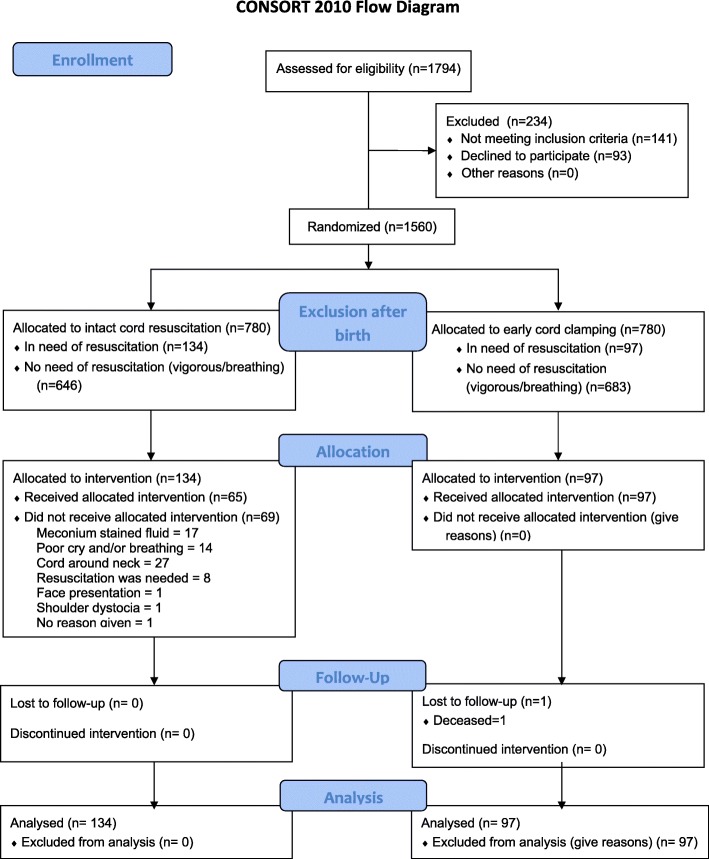
Trial profile. Flow diagram adapted from the CONSORT flow diagram (http://www.consort-statement.org/)

From April 20 to August 27, 2016, 1560 women who were admitted to MNSC agreed to participate in the study. After signed consent was obtained and as birth was imminent, 780 women were randomly assigned to early cord clamping and 780 to delayed cord clamping where CC occurred after at least 3 minutes subsequent to the appearance of the infant’s shoulder. Of the 1560 included newborns, 1329 were breathing and were excluded, while 231 were assessed as needing resuscitation according to the HBB algorithm (e.g. not crying), 141 boys (61.0%) and 90 girls (39.0%); mean (SD) gestational age, 39.6 (1.4) weeks. More infants were assessed as needing resuscitation in the delayed CC group, 134 (17.2%) compared to 97 (12.4%) in the early CC group. In the power analysis we expected even groups and aimed for a group size of 99 after attrition. The difference was consistent through the recruitment period. The allocated intervention was followed in 65 (48.6%) cases in the intact cord group in contrast to 97 (100%) in the early CC group (Fig. 3). Lowest adherence to protocol in the delayed CC group was in April; 4 cases (25.0%) and highest in July: 28 cases (70.0%) but fell again in August: 6 (33.3%). Deliveries were distributed with a median (min to max range) of 27 (16 to 40) newborns per fortnight, and evenly throughout the day; 58 (25%) during the first 6 h, 47 (20%), during the next six until noon, 57 (25%) the 6 h after noon and 69 (30%) last 6 h until midnight. Clearing of airways was sufficient to achieve normal breathing in 35 (15%) infants, 74 (32%) needed stimulation, and bag and mask ventilation had to be performed on 122 (53%) newborns. The median time to clamping the umbilical cord was 105 (interquartile range, 30–191) seconds for the intact cord group and 25 (interquartile range, 14–40) seconds for the early clamping group (*P* < .001). Breathing attempts were observed before clamping the cord in 88 (65.7%) newborns in the intact cord group, compared to 19 (19.6%) in the early CC group, *P* < 0.001. We found some significant differences between the delayed and early clamping groups with respect to maternal characteristics: mothers in the intact cord group were slightly older and had a higher mean number of pregnancies. Neither mothers age or number of pregnancies were significantly correlated to any of the primary or secondary outcomes. Neonatal baseline data such as gestational age and birth weight were similar between groups as was presence of meconium stained amniotic fluid (Table 1).

**Table 1:** Baseline characteristics of mothers and newborn infants who were randomized to resuscitation with an intact cord (intervention) or after early cord clamping (control)

	Cord clamping group	
	Intact cord > 3 min (*n* = 134)	Early < 1 min (*n* = 97)
Maternal characteristics		
Maternal age, years^a^	23.2 (4.1)	22.1 (3.5)
Parity (including study child)^b^	1.5 (0.8)	1.3 (0.6)
Vaginal delivery (non-instrumental)	134 (100%)	97 (100%)
Infant characteristics		
Gestational age, weeks	39.6 (1.4)	39.6 (1.4)
Male sex	77 (57%)	64 (66%)
Meconium stained amniotic fluid	58 (43.3%)	40 (41.2%)
Birth weight, g	3072 (401)	3036 (372)

Data are mean (SD) or n (%)

^a^Difference 1.1 years (95% CI 0.1–2.1), *P* value = .03

^b^Difference 0.2 children (95% CI 0.0–0.4), *P* value = .02

### Primary outcome

**Fig. 4:**
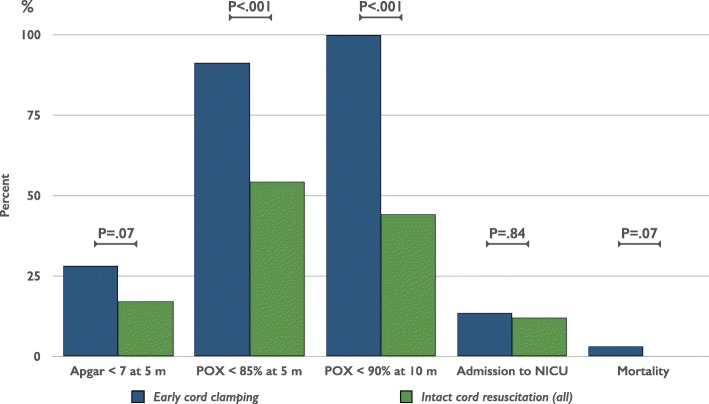
Outcome on categorical variables after resuscitation with an intact cord versus early cord clamping. *P*-value calculated using Fischer’s exact test

At 10 min after birth, oxygen saturation was significantly higher (5.0%) in the intact cord group compared to the early CC group (Table 2). In the intact cord group, 57 (44%) had oxygen saturation < 90% after 10 min, compared to 93 (100%) in the early CC group, absolute risk reduction 56% (CI 48–56%), numbers needed to treat 1.8 (CI 1.8–2.1) (Fig. 4).

**Table 2:** Measurements from infants who were randomized to resuscitation with an intact cord (intervention) or after early cord clamping (control). Intention to treat analysis

	Cord Clamping Group, Mean (SD)		Difference (95% CI)	*P* value^a^
	Intact cord > 3 min	Early < 1 min)		
Readings from pulse oximeter	(*n* = 129)	(*n* = 93)		
Saturation at 1 min (%)	71.5 (9.3)	62.4 (4.3)	9.1 (7.3 to 11.0)	<.001
Saturation at 5 min (%)	83.6 (8.3)	76.6 (4.1)	7.0 (5.3 to 8.7)	<.001
Saturation at 10 min (%)	90.4 (8.1)	85.4 (2.7)	5.0 (3.5 to 6.5)	<.001
Heart rate at 1 min	105 (3)	116 (5)	−10 (− 11 to −9)	<.001
Heart rate at 5 min	124 (4)	134 (4)	−10 (− 11 to − 8)	<.001
Heart rate at 10 min	136 (2)	135 (2)	1 (0 to 1)	.03
Apgar score	(*n* = 134)	(*n* = 97)		
Apgar at 1 min	5.1 (1.0)	4.3 (1.0)	0.8 (0.5 to 1.1)	<.001
Apgar at 5 min	6.8 (0.7)	6.5 (1.1)	0.3 (0.1 to 0.5)	.01
Apgar at 10 min	9.4 (1.0)	9.0 (1.5)	0.4 (0.2 to 0.7)	.03
Timed events, Median (interquartile range)	(*n* = 134)	(*n* = 97)		
Time of start of resuscitation (sec)	45 (28 to 68)	45 (25 to 59)		. 19^b^
Time of first cry/breath (sec)	37 (34 to 44)	45 (38 to 50)		<.001^b^
Time of regular breathing (sec)^c^	78 (67 to 96)	98 (89 to 356)		<.001^b^
Time to cord clamping (sec)	104 (30 to 191)	25 (14 to 40)		<.001^b^
Measurements after resuscitation				
Baby’s temperature (C°)^d^	36.2 (0.5)	36.2 (0.5)	0.0 (−0.1 to 0.1)	.82
Transcutaneous bilirubin (μmol/L) at discharge^e^	97.8 (52.5)	89.1 (52.6)	8.6 (−5.5 to 23.0)	.23

^a^Calculated using an unpaired 2-tailed t test

^b^Calculated using Mann Whitney U test

^c^Intact cord group *n* = 132 and the early group *n* = 93

^d^Intact cord group *n* = 127 and the early group *n* = 90

^e^Age (hours) at discharge was median (interquartile range) 21.6 (15.4 to 31.6) in the intact cord group (*n* = 128) vs 22.2 (14.5 to 33.9) in the early group (*n* = 95), *p* = .83

### Secondary outcomes

Oxygen saturation was also significantly higher in the intact cord group at one and five minutes after birth. At 5 min, the intact cord group, 70 (54%) had oxygen saturation < 85% compared to 85 (91%) in the early CC group, absolute risk reduction 37% (26 to 45%), *P* < .001, numbers needed to treat 2.7 (CI 2.2–3.9). (Fig. 4).

Heart rate was lower in the intact cord group at one and five minutes and slightly higher at ten minutes, all significant findings (Table 2). Apgar score was significantly higher at one, five and ten minutes. At 5 min, 23 (17%) had Apgar score < 7 in the intact cord group compared to 26 (27%) in the early CC group, absolute risk reduction 10% (− 2 to 21%), *P* < .07. (Fig. 4).

Time of initiating resuscitation was later in the intact cord group while newborns started breathing and established regular breathing earlier (Table 2). Temperature was measured at mean (SD) 18.0 (7.1) minutes, and there was no difference in temperature between groups. Also, transcutaneous bilirubin measured at discharge did not differ. (Table 2).

In the intact cord group, 16 (11.9%) newborns were transferred to the Neonatal intensive care unit, not significantly different from 13 (13.4%) in the early CC group, *P* = .84. The mortality rate before discharge was 0 (0.0%) in the intact cord resuscitation group versus 3 (3.1%) in the early CC group, *P* = .07. (Fig. 4).

When analysis was performed including only newborns handled according to protocol, results were similar, or more pronounced (Table 3). Also, when analysis was performed including only those newborns requiring bag and mask resuscitation, results were similar (Table 4). An ANOVA analysis including post-hoc multiple comparisons comparing the infants divided into three groups, intact cord resuscitation per protocol (CC ≥ 180 s), intact cord resuscitation protocol violations (CC < 180 s) and early CC did demonstrate that early CC was similar or disadvantageous compared to the two intact cord resuscitation groups [see Additional file 1].

**Table 3:** Measurements from infants who were randomized to resuscitation with an intact cord (intervention) or after early cord clamping (control). Only infants handled according to allocation (per protocol) analyzed

	Cord Clamping Group, Mean (SD)		Difference (95% CI)	*P* value^a^
	Intact cord > 3 min	Early < 1 min		
Readings from pulse oximeter	(*n* = 63)	(*n* = 93)		
Saturation at 1 min (%)	79.8 (3.6)	62.4 (4.3)	17.4 (16.1 to 18.7)	<.001
Saturation at 5 min (%)	91.4 (3.1)	76.6 (4.1)	14.8 (13.6 to 16.0)	<.001
Saturation at 10 min (%)	98.0 (1.4)	85.4 (2.7)	12.6 (11.8 to 13.3)	<.001
Heart rate at 1 min	105 (3)	116 (5)	−11 (−12 to −10)	<.001
Heart rate at 5 min	125 (3)	134 (4)	−9 (−10 to −7)	<.001
Heart rate at 10 min	137 (2)	135 (2)	2 (1 to 2)	<.001
Apgar score	(*n* = 63)	(*n* = 93)		
Apgar at 1 min	5.7 (0.9)	4.3 (0.9)	1.4 (1.1 to 1.6)	<.001
Apgar at 5 min	7.0 (0.5)	6.6 (0.9)	0.5 (0.2 to 0.7)	<.001
Apgar at 10 min	9.6 (0.7)	9.0 (1.3)	0.6 (0.2 to 1.0)	.001
Timed events, Median (interquartile range)	(*n* = 65)	(*n* = 97)		
Time of start of resuscitation (sec)	40 (22 to 60)	45 (25 to 59)		.62^b^
Time of first cry/breath (sec)	34 (28 to 39)	45 (38 to 50)		<.001^b^
Time of regular breathing (sec)^c^	69 (34 to 399)	98 (89 to 356)		<.001^b^
Time to cord clamping (sec)	192 (188 to 200)	25 (14 to 40)		<.001^b^
Measurements after resuscitation				
Baby’s temperature (C°) 30 min after birth^d^	36.2 (0.6)	36.2 (0.5)	0.0 (−0.2 to 0.2)	.82
Transcutaneous bilirubin (μmol/L) at discharge^e^	94.4 (44.1)	89.1 (52.6)	5.3 (−11.0 to 21.7)	.52

^a^Calculated using an unpaired 2-tailed t test

^b^Calculated using Mann Whitney U test

^c^Intact cord group *n* = 65 and the early group *n* = 93

^d^Intact cord group *n* = 64 and the early group *n* = 90

^e^Age (hours) at discharge was median (interquartile range) 22.5 (16.2 to 32.1) in the intact cord group (*n* = 61) vs 22.2 (14.5 to 33.9) in the early group (*n* = 95), *p* = .81

**Table 4:** Measurements from infants who were randomized to resuscitation with an intact cord (intervention) or after early cord clamping (control), only including those resuscitated with bag and mask

	Cord Clamping Group, Mean (SD)		Difference (95% CI)	*P*-value^a^
	Intact cord > 3 min	Early < 1 min)		
Readings from pulse oximeter	(*n* = 72)	(*n* = 45)		
Saturation at 1 min (%)	73.7 (8.8)	62.6 (5.0)	11.1 (8.2 to 13.9)	<.001
Saturation at 5 min (%)	85.6 (8.1)	76.1 (3.9)	9.4 (6.9 to 12.0)	<.001
Saturation at 10 min (%)	91.9 (7.9)	85.1 (2.8)	6.8 (4.4 to 9.3)	<.001
Heart rate at 1 min	106 (3)	115 (5)	−10 (−11 to −8)	<.001
Heart rate at 5 min	124 (4)	134 (4)	−10 (− 11 to −8)	<.001
Heart rate at 10 min	136 (2)	136 (2)	1 (0 to 2)	.05
Apgar score	(*n* = 74)	(*n* = 48)		
Apgar at 1 min	5.0 (1.1)	3.8 (1.1)	1.2 (0.8 to 1.6)	< 0.001
Apgar at 5 min	6.7 (0.9)	6.0 (1.3)	0.7 (0.3 to 1.1)	0.001
Apgar at 10 min	9.2 (1.2)	8.3 (1.8)	0.9 (0.3 to 1.5)	0.005
Timed events, Median (interquartile range)	(*n* = 74)	(*n* = 48)		
Time of start of resuscitation (sec)	48 (30 to 71)	43 (23 to 57)		. 06^b^
Time of first cry/breath (sec)	38 (34 to 45)	48 (45 to 62)		<.001^b^
Time of regular breathing (sec)^c^	78 (67 to 155)	356 (98 to 389)		<.001^b^
Time to cord clamping (sec)	187 (42 to 195)	25 (11 to 40)		<.001 ^b^

^a^Calculated using an unpaired 2-tailed t test

^b^Calculated using Mann Whitney U test

^c^Intact cord group *n* = 72 and the early group *n* = 45

## Discussion

In this trial, where randomization was performed while the baby was still in utero, resuscitation measures performed according to the HBB algorithm with an unclamped cord in the mother’s bed were associated with a higher oxygen saturation and a higher Apgar score at one, five and ten minutes. Breathing was initiated earlier, and no signs of harm were found, when compared to clamping and cutting the cord before initiation of resuscitation measures. In contrast to experimental animal studies, but in congruence with human studies, heart rate was lower at one and five minutes after intact cord, compared to early CC [15, 20]. A low rate of protocol adherence affects the generalizability of the results.

In their important study on preterm lambs published in 2013, Bhatt et al. could demonstrate a more stable cardiovascular adaptation if cord clamping was performed after initiation of ventilation [15]. In the Bhatt et al. study, heart rate dropped during the first 90 s if the cord was clamped before ventilating while in our study as well as in the observational study by Smit et al. on human newborns, heart rate was initially lower in the delayed CC group. We hypothesize that a smoother transition ensures a higher blood volume and a larger heart stroke volume, allowing for a lower heart rate with maintained cardiac output. The results from these, and other studies rendered several papers suggesting that resuscitation of newborns should be performed with an uncut cord, facilitating the postnatal transition [17, 18, 27]. The increased oxygenation found in our study may be due to several interacting mechanisms; a persisting oxygenation by the placenta, an increased pulmonary blood flow, earlier initiation of breathing, and an improved perfusion of organs and peripheral tissue including skin and muscles due to optimized blood volume and pressure. To our knowledge, resuscitation with an uncut cord has only been studied in humans in a few studies. Katheria et al. conducted a feasibility study of 60 term newborns that were at risk of resuscitation, randomized to either 1 minute or 5 minute CC [21]. Only 53% (*n* = 32) actually needed resuscitation but the findings in the study did show greater cerebral oxygenation and blood pressure in the 5-minute group where resuscitation was performed with an intact placental circulation. In concordance to our study, SpO2 was higher in the 5-minute CC group, although only significant at three and four minutes in subjects not requiring supplemental oxygen or positive pressure ventilation. Also, time to first breath/cry was similar to our findings, although not significant; mean (SD) 25 (42) in the 5-minute group compared to 36 (61) in the 1-minute group [21]. In contrast, Ersdal et al. could not demonstrate any relationship between time to CC and onset of breathing or initiation of positive pressure ventilation following stimulation/suction, or 24-h outcome in an observational study [28]. In agreement with our results that intact cord resuscitation might be favorable, Lefebvre et al. studied newborns that had in utero congenital diaphragmatic hernia in an observational study, and could demonstrate that an initiation of resuscitation measures before clamping the umbilical cord was associated with higher pH and lower plasma lactate at 1 h after birth, as well as higher blood pressure at one, six and 12 h after birth [29].

Our study suffers from certain limitations. There was a high degree of protocol violation in the intact cord group, and it is possible that the newborns among the protocol violation group were in a more depressed state than those remaining in the intact cord resuscitation group. Prenatal fetal monitoring as well as blood gas sampling from the umbilical cord might have given a better understanding regarding this issue. A separate analysis comparing outcomes between the subgroup of infants randomized to intact cord resuscitation that had their cord cut before 180 s (protocol violators) to the early CC group could not demonstrate any negative effects in the protocol violator group. An extensive education and training program was performed involving staff and research team members, but this was not enough to avoid protocol violations, even if staff were accustomed to delayed CC after an earlier study at the same hospital [10]. We examined if the protocol adherence rate varied according to hour of the day and by months but could not find any such pattern. Proposed steps for implementing delayed cord clamping have been published [30]. Due to sparse resources regarding post-natal clinical care, the study also lacks clinical data. Data were collected manually from the screen from the oximeter, resulting in a risk for research team members to unconsciously pick a higher number for one group and a lower number for the other. In this study, masking was not possible.

This study provides new and important information on the effects of resuscitation with an intact cord: In the group allocated to having their cord clamped and cut after three minutes, newborns had higher SpO_2_ and higher Apgar score, and no negative consequences were recorded, similar to reports on preterm infants [31]. Resuscitation with an intact cord do not need investments in advanced equipment, in agreement with other approaches that are less interventional to their character, such as giving ventilation with air instead of 100% oxygen and caring of low birth weight infants skin to skin [32, 33]. These approaches are especially, but not exclusively, feasible in low resource settings suffering from high perinatal mortality and morbidity [5].

## Conclusions

We conclude that in term infants, resuscitation with an intact umbilical cord did not raise any safety concerns and was associated with a better recovery than routine resuscitation after clamping and cutting the cord, in concordance with experimental studies. Before implementing in recommendations from ILCOR and algorithms as HBB, further studies are needed to verify our findings in settings where more detailed prenatal and postnatal surveillance is possible, as well as including larger numbers to explore the implied effects on decreased admission rate to neonatal care and mortality and to pursue long-term neurodevelopmental follow up.

## Additional file

Additional file 1: ANOVA analysis including post-hoc multiple comparisons comparing the infants divided into three groups, intact cord resuscitation per protocol (CC ≥ 180 s), intact cord resuscitation protocol violaton (CC < 180 s) and early CC. (PDF 61 kb)

## Data Availability

The datasets used and/or analyzed during the current study are available from the corresponding author on reasonable request.

## Abbreviations

CC: Cord clamping
HBB: Helping Babies Breathe, a simplified algorithm for neonatal resuscitation
ILCOR: International Liaison Committee on Resuscitation
MNSC: Maternal and Neonatal Service Centre
SpO_2_: Saturation by pulse oximetry
