# Supplementary material for: Intact cord resuscitation versus early cord clamping in the treatment of depressed newborn infants during the first 10 minutes of birth (Nepcord III) – a randomized clinical trial
Source: Matern Health Neonatol Perinatol. 2019 Aug 29;5:15. doi: 10.1186/s40748-019-0110-z (PMC6714434; doi:10.1186/s40748-019-0110-z)
Supplement: Supplementary file 1 — ANOVA analysis including post-hoc multiple comparisons comparing the infants divided into three groups, intact cord resuscitation per protocol (CC ≥ 180 s), intact cord resuscitation protocol violaton (CC < 180 s) and early CC. (PDF 61 kb) [file 40748_2019_110_MOESM1_ESM.pdf]

Descriptives

		N	Mean	Std. Deviation	Std. Error	95% Confidence Interval for Mean Lower Bound	Upper Bound	Minimum	Maximum
Gestational age	Early	97	39,63	1,3782	0,1399	39,353	39,908	35,1	42
	ICR group DCC < 180 s (protocol breaker)	69	39,741	1,358	0,1635	39,415	40,067	35,4	42
	ICR group DCC >= 180 (per protocol)	65	39,387	1,4213	0,1763	39,035	39,739	34,9	41,6
	Total	231	39,595	1,3854	0,0912	39,415	39,775	34,9	42
Birth weight (grams)	Early	95	3036,53	372,33	38,2	2960,68	3112,37	1920	4000
	ICR group DCC < 180 s (protocol breaker)	69	3054,93	425,245	51,194	2952,77	3157,08	2060	4120
	ICR group DCC >= 180 (per protocol)	65	3090,38	377,069	46,77	2996,95	3183,82	2230	4080
	Total	229	3057,36	389,219	25,72	3006,68	3108,04	1920	4120
Time to umbilical cord was clamped	Early	97	26,13	17,064	1,733	22,69	29,57	0	59
	ICR group DCC < 180 s (protocol breaker)	69	33,68	29,186	3,514	26,67	40,69	0	126
	ICR group DCC >= 180 (per protocol)	65	195,54	15,099	1,873	191,8	199,28	180	282
	Total	231	76,06	77,853	5,122	65,96	86,15	0	282
Time of POX placement	Early	93	40,05	4,73	0,491	39,08	41,03	28	49
	ICR group DCC < 180 s (protocol breaker)	66	40,52	4,638	0,571	39,37	41,66	30	49
	ICR group DCC >= 180 (per protocol)	63	41,24	5,044	0,635	39,97	42,51	30	49
	Total	222	40,53	4,798	0,322	39,89	41,16	28	49
POX Saturation reading at 1 minute	Early	93	62,41	4,339	0,45	61,51	63,3	45	69
	ICR group DCC < 180 s (protocol breaker)	66	63,62	5,241	0,645	62,33	64,91	47	74
	ICR group DCC >= 180 (per protocol)	63	79,79	3,633	0,458	78,88	80,71	68	86
	Total	222	67,7	8,835	0,593	66,53	68,87	45	86
POX Saturation reading at 5 minute	Early	93	76,62	4,128	0,428	75,77	77,47	64	87
	ICR group DCC < 180 s (protocol breaker)	66	76,18	3,142	0,387	75,41	76,95	66	83
	ICR group DCC >= 180 (per protocol)	63	91,44	3,068	0,387	90,67	92,22	80	94
	Total	222	80,7	7,657	0,514	79,69	81,71	64	94
POX Saturation reading at 10 minute	Early	93	85,4	2,715	0,282	84,84	85,96	77	89
	ICR group DCC < 180 s (protocol breaker)	66	83,09	4,139	0,509	82,07	84,11	77	90
	ICR group DCC >= 180 (per protocol)	63	97,98	1,431	0,18	97,62	98,34	95	100
	Total	222	88,28	6,86	0,46	87,38	89,19	77	100
POX Heart rate reading at 1 minute	Early	93	115,74	4,845	0,502	114,74	116,74	104	132
	ICR group DCC < 180 s (protocol breaker)	66	106,18	3,215	0,396	105,39	106,97	101	121
	ICR group DCC >= 180 (per protocol)	63	104,75	2,885	0,363	104,02	105,47	100	111
	Total	222	109,78	6,418	0,431	108,93	110,63	100	132
POX Heart rate reading at 5 minute	Early	93	133,62	4,115	0,427	132,78	134,47	121	140
	ICR group DCC < 180 s (protocol breaker)	66	123,18	4,472	0,55	122,08	124,28	117	135
	ICR group DCC >= 180 (per protocol)	63	125,05	2,964	0,373	124,3	125,79	120	134
	Total	222	128,09	6,174	0,414	127,27	128,9	117	140
POX Heart rate reading at 10 minute	Early	93	135,44	2,357	0,244	134,96	135,93	127	139
	ICR group DCC < 180 s (protocol breaker)	66	135,36	2,237	0,275	134,81	135,91	126	139
	ICR group DCC >= 180 (per protocol)	63	136,98	2,218	0,279	136,43	137,54	131	140
	Total	222	135,86	2,381	0,16	135,54	136,17	126	140
Apgar 1 min	Early	97	4,27	1,026	0,104	4,06	4,47	0	6
	ICR group DCC < 180 s (protocol breaker)	69	4,52	0,797	0,096	4,33	4,71	2	6
	ICR group DCC >= 180 (per protocol)	65	5,68	0,85	0,105	5,47	5,89	4	7
	Total	231	4,74	1,088	0,072	4,6	4,88	0	7
Apgar 5 min	Early	97	6,51	1,032	0,105	6,3	6,71	2	8
	ICR group DCC < 180 s (protocol breaker)	69	6,61	0,79	0,095	6,42	6,8	4	8

	ICR group DCC >= 180 (per protocol)	65	7,02	0,515	0,064	6,89	7,14	5	8
	Total	231	6,68	0,866	0,057	6,57	6,79	2	8
Apgar 10 min	Early	96	8,97	1,49	0,152	8,67	9,27	2	10
	ICR group DCC < 180 s (protocol breaker)	69	9,1	1,19	0,143	8,82	9,39	5	10
	ICR group DCC >= 180 (per protocol)	65	9,65	0,738	0,092	9,46	9,83	7	10
	Total	230	9,2	1,255	0,083	9,04	9,36	2	10
Baby's Temperature (Celcius)	Early	90	36,1951	0,4951	0,05219	36,0914	36,2988	34,61	37,06
	ICR group DCC < 180 s (protocol breaker)	66	36,2012	0,50813	0,06255	36,0763	36,3261	35,11	37,67
	ICR group DCC >= 180 (per protocol)	64	36,1753	0,55496	0,06937	36,0367	36,314	34,61	37,61
	Total	220	36,1912	0,51474	0,0347	36,1228	36,2596	34,61	37,67
Transcutaneous bilirubin measured at discharge	Early	91	89,08	52,617	5,516	78,12	100,04	18	321
	ICR group DCC < 180 s (protocol breaker)	68	100,79	58,998	7,155	86,51	115,07	21	335
	ICR group DCC >= 180 (per protocol)	59	94,42	44,093	5,74	82,93	105,91	1	222
	Total	218	94,18	52,605	3,563	87,16	101,2	1	335

ANOVA

		Sum of Square	df	Mean Square	F	Sig.
Gestational age	Between Groups	4,413	2	2,207	1,151	0,318
	Within Groups	437,051	228	1,917		
	Total	441,464	230			
Birth weight (grams)	Between Groups	112532,931	2	56266,466	0,369	0,692
	Within Groups	34427443,7	226	152333,822		
	Total	34539976,6	228			
Time to umbilical cord was clamped	Between Groups	1293585,87	2	646792,936	1467,842	<0.001
	Within Groups	100466,397	228	440,642		
	Total	1394052,27	230			
Time of POX placement	Between Groups	52,693	2	26,347	1,146	0,32
	Within Groups	5034,645	219	22,989		
	Total	5087,338	221			
POX Saturation reading at 1 minute	Between Groups	12916,058	2	6458,029	326,154	<0.001
	Within Groups	4336,321	219	19,801		
	Total	17252,378	221			
POX Saturation reading at 5 minute	Between Groups	10165,578	2	5082,789	398,514	<0.001
	Within Groups	2793,202	219	12,754		
	Total	12958,779	221			
POX Saturation reading at 10 minute	Between Groups	8482,403	2	4241,202	484,085	<0.001
	Within Groups	1918,718	219	8,761		
	Total	10401,122	221			
POX Heart rate reading at 1 minute	Between Groups	5756,624	2	2878,312	188,301	<0.001
	Within Groups	3347,561	219	15,286		
	Total	9104,185	221			
POX Heart rate reading at 5 minute	Between Groups	5020,871	2	2510,435	161,583	<0.001
	Within Groups	3402,503	219	15,537		
	Total	8423,374	221			
POX Heart rate reading at 10 minute	Between Groups	112,206	2	56,103	10,767	<0.001
	Within Groups	1141,182	219	5,211		
	Total	1253,387	221			
Apgar 1 min	Between Groups	81,952	2	40,976	49,051	<0.001
	Within Groups	190,464	228	0,835		
	Total	272,416	230			
Apgar 5 min	Between Groups	10,628	2	5,314	7,494	0,001
	Within Groups	161,667	228	0,709		
	Total	172,294	230			
Apgar 10 min	Between Groups	18,742	2	9,371	6,219	0,002
	Within Groups	342,058	227	1,507		
	Total	360,8	229			
Baby's Temperature (Celcius)	Between Groups	0,024	2	0,012	0,045	0,956
	Within Groups	58,001	217	0,267		
	Total	58,025	219			

Transcutaneous bilirubin measured at discharge	Between Groups	5348,037	2	2674,018	0,966	0,382
	Within Groups	595151,986	215	2768,149		
	Total	600500,023	217			

Post Hoc Tests
Multiple Comparisons
Bonferroni

Dependent Variable	(I) Studygroup_pp_3	(J) Studygroup	Mean Differen	Std. Error	Sig.	95% Confidence Interval	
						Lower Bound	Upper Bound
Gestational age	Early	ICR group DC(	-0,1109	0,218	>0.99	-0,637	0,415
		ICR group DC(	0,2435	0,2219	0,821	-0,292	0,779
	ICR group DCC < 180 s (protocol breaker)	Early	0,1109	0,218	>0.99	-0,415	0,637
		ICR group DC(	0,3544	0,2393	0,42	-0,223	0,932
	ICR group DCC >= 180 (per protocol)	Early	-0,2435	0,2219	0,821	-0,779	0,292
		ICR group DC(	-0,3544	0,2393	0,42	-0,932	0,223
Birth weight (grams)	Early	ICR group DC(	-18,401	61,735	>0.99	-167,3	130,5
		ICR group DC(	-53,858	62,826	>0.99	-205,39	97,67
	ICR group DCC < 180 s (protocol breaker)	Early	18,401	61,735	>0.99	-130,5	167,3
		ICR group DC(	-35,457	67,464	>0.99	-198,17	127,26
	ICR group DCC >= 180 (per protocol)	Early	53,858	62,826	>0.99	-97,67	205,39
		ICR group DC(	35,457	67,464	>0.99	-127,26	198,17
Time to umbilical cord was clamped	Early	ICR group DC(	-7,547	3,306	0,07	-15,52	0,43
		ICR group DC(	-169,404*	3,365	<0.001	-177,52	-161,29
	ICR group DCC < 180 s (protocol breaker)	Early	7,547	3,306	0,07	-0,43	15,52
		ICR group DC(	-161,857*	3,628	<0.001	-170,61	-153,11
	ICR group DCC >= 180 (per protocol)	Early	169,404*	3,365	<0.001	161,29	177,52
		ICR group DC(	161,857*	3,628	<0.001	153,11	170,61
Time of POX placement	Early	ICR group DC(	-0,461	0,772	>0.99	-2,32	1,4
		ICR group DC(	-1,184	0,782	0,395	-3,07	0,7
	ICR group DCC < 180 s (protocol breaker)	Early	0,461	0,772	>0.99	-1,4	2,32
		ICR group DC(	-0,723	0,845	>0.99	-2,76	1,31
	ICR group DCC >= 180 (per protocol)	Early	1,184	0,782	0,395	-0,7	3,07
		ICR group DC(	0,723	0,845	>0.99	-1,31	2,76
POX Saturation reading at 1 minute	Early	ICR group DC(	-1,213	0,716	0,276	-2,94	0,52
		ICR group DC(	-17,385*	0,726	<0.001	-19,14	-15,63
	ICR group DCC < 180 s (protocol breaker)	Early	1,213	0,716	0,276	-0,52	2,94
		ICR group DC(	-16,172*	0,784	<0.001	-18,06	-14,28
	ICR group DCC >= 180 (per protocol)	Early	17,385*	0,726	<0.001	15,63	19,14
		ICR group DC(	16,172*	0,784	<0.001	14,28	18,06
POX Saturation reading at 5 minute	Early	ICR group DC(	0,442	0,575	>0.99	-0,94	1,83
		ICR group DC(	-14,821*	0,583	<0.001	-16,23	-13,41
	ICR group DCC < 180 s (protocol breaker)	Early	-0,442	0,575	>0.99	-1,83	0,94
		ICR group DC(	-15,263*	0,629	<0.001	-16,78	-13,75
	ICR group DCC >= 180 (per protocol)	Early	14,821*	0,583	<0.001	13,41	16,23
		ICR group DC(	15,263*	0,629	<0.001	13,75	16,78
POX Saturation reading at 10 minute	Early	ICR group DC(	2,307*	0,476	<0.001	1,16	3,46
		ICR group DC(	-12,586*	0,483	<0.001	-13,75	-11,42
	ICR group DCC < 180 s (protocol breaker)	Early	-2,307*	0,476	<0.001	-3,46	-1,16
		ICR group DC(	-14,893*	0,521	<0.001	-16,15	-13,64
	ICR group DCC >= 180 (per protocol)	Early	12,586*	0,483	<0.001	11,42	13,75
		ICR group DC(	14,893*	0,521	<0.001	13,64	16,15
POX Heart rate reading at 1 minute	Early	ICR group DC(	9,560*	0,629	<0.001	8,04	11,08
		ICR group DC(	10,996*	0,638	<0.001	9,46	12,53
	ICR group DCC < 180 s (protocol breaker)	Early	-9,560*	0,629	<0.001	-11,08	-8,04
		ICR group DC(	1,436	0,689	0,115	-0,23	3,1

	ICR group DCC >= 180 (per protocol)	Early	-10,996*	0,638	<0.001	-12,53	-9,46
		ICR group DC(	-1,436	0,689	0,115	-3,1	0,23
POX Heart rate reading at 5 minute	Early	ICR group DC(	10,442*	0,634	<0.001	8,91	11,97
		ICR group DC(	8,576*	0,643	<0.001	7,02	10,13
	ICR group DCC < 180 s (protocol breaker)	Early	-10,442*	0,634	<0.001	-11,97	-8,91
		ICR group DC(	-1,866*	0,694	0,023	-3,54	-0,19
	ICR group DCC >= 180 (per protocol)	Early	-8,576*	0,643	<0.001	-10,13	-7,02
		ICR group DC(	1,866*	0,694	0,023	0,19	3,54
POX Heart rate reading at 10 minute	Early	ICR group DC(	0,077	0,367	>0.99	-0,81	0,96
		ICR group DC(	-1,543*	0,372	<0.001	-2,44	-0,64
	ICR group DCC < 180 s (protocol breaker)	Early	-0,077	0,367	>0.99	-0,96	0,81
		ICR group DC(	-1,620*	0,402	<0.001	-2,59	-0,65
	ICR group DCC >= 180 (per protocol)	Early	1,543*	0,372	<0.001	0,64	2,44
		ICR group DC(	1,620*	0,402	<0.001	0,65	2,59
Apgar 1 min	Early	ICR group DC(	-0,254	0,144	0,238	-0,6	0,09
		ICR group DC(	-1,409*	0,147	<0.001	-1,76	-1,06
	ICR group DCC < 180 s (protocol breaker)	Early	0,254	0,144	0,238	-0,09	0,6
		ICR group DC(	-1,155*	0,158	<0.001	-1,54	-0,77
	ICR group DCC >= 180 (per protocol)	Early	1,409*	0,147	<0.001	1,06	1,76
		ICR group DC(	1,155*	0,158	<0.001	0,77	1,54
Apgar 5 min	Early	ICR group DC(	-0,104	0,133	>0.99	-0,42	0,22
		ICR group DC(	-,510*	0,135	0,001	-0,84	-0,18
	ICR group DCC < 180 s (protocol breaker)	Early	0,104	0,133	>0.99	-0,22	0,42
		ICR group DC(	-,407*	0,146	0,017	-0,76	-0,06
	ICR group DCC >= 180 (per protocol)	Early	,510*	0,135	0,001	0,18	0,84
		ICR group DC(	,407*	0,146	0,017	0,06	0,76
Apgar 10 min	Early	ICR group DC(	-0,133	0,194	>0.99	-0,6	0,33
		ICR group DC(	-,677*	0,197	0,002	-1,15	-0,2
	ICR group DCC < 180 s (protocol breaker)	Early	0,133	0,194	>0.99	-0,33	0,6
		ICR group DC(	-,545*	0,212	0,033	-1,06	-0,03
	ICR group DCC >= 180 (per protocol)	Early	,677*	0,197	0,002	0,2	1,15
		ICR group DC(	,545*	0,212	0,033	0,03	1,06
Baby's Temperature (Celcius)	Early	ICR group DC(	-0,00612	0,08378	>0.99	-0,2083	0,196
		ICR group DC(	0,01971	0,08454	>0.99	-0,1842	0,2237
	ICR group DCC < 180 s (protocol breaker)	Early	0,00612	0,08378	>0.99	-0,196	0,2083
		ICR group DC(	0,02583	0,0907	>0.99	-0,193	0,2447
	ICR group DCC >= 180 (per protocol)	Early	-0,01971	0,08454	>0.99	-0,2237	0,1842
		ICR group DC(	-0,02583	0,0907	>0.99	-0,2447	0,193
Transcutaneous bilirubin measured at discharge	Early	ICR group DC(	-11,717	8,434	0,499	-32,07	8,63
		ICR group DC(	-5,347	8,794	>0.99	-26,57	15,87
	ICR group DCC < 180 s (protocol breaker)	Early	11,717	8,434	0,499	-8,63	32,07
		ICR group DC(	6,37	9,361	>0.99	-16,22	28,96
	ICR group DCC >= 180 (per protocol)	Early	5,347	8,794	>0.99	-15,87	26,57
		ICR group DC(	-6,37	9,361	>0.99	-28,96	16,22

* The mean difference is significant at the 0.05 level.
